# Identification of a Prognostic Transcriptome Signature for Hepatocellular Carcinoma with Lymph Node Metastasis

**DOI:** 10.1155/2022/7291406

**Published:** 2022-07-06

**Authors:** Jie Ma, Xue-Qin Chen, Zuo-Lin Xiang

**Affiliations:** ^1^Department of Radiation Oncology, Shanghai East Hospital, School of Medicine, Tongji University, Shanghai 200120, China; ^2^Department of Radiation Oncology, Shanghai East Hospital Ji'an hospital, Ji'an City, 343000, Jiangxi Province, China

## Abstract

Hepatocellular carcinoma (HCC) is one of the most aggressive malignant tumors, and the prognosis of HCC patients with lymph node metastasis (LNM) is poor. However, robust biomarkers for predicting the prognosis of HCC LNM are still lacking. This study used weighted gene coexpression network analysis of GSE28248 (*N* = 80) microarray data to identify gene modules associated with HCC LNM and validated in GSE40367 dataset (*N* = 18). The prognosis-related genes in the HCC LNM module were further screened based on the prognostic curves of 371 HCC samples from TCGA. We finally developed a prognostic signature, PSG-30, as a prognostic-related biomarker in HCC LNM. The HCC subtypes identified by PSG-30-based consensus clustering analysis showed significant differences in prognosis, clinicopathological stage, m6A modification, ferroptosis activation, and immune characteristics. In addition, *RAD54B* was selected by regression model as an independent risk factor affecting the prognosis of HCC patients with LNM, and its expression was significantly positively correlated with tumor mutational burden and microsatellite instability in high-risk subtypes. Patients with high *RAD54B* expression had a better prognosis in the immune checkpoint inhibitor-treated cohorts but had a poor prognosis in the HCC sorafenib-treated group. The association of high *RAD54B* expression with LNM in breast cancer (BRCA) and cholangiocarcinoma and its prognostic effect in BRCA LNM cases suggest the value of *RAD54B* at the pancancer level. In conclusion, PSG-30 can effectively identify HCC LNM population with poor prognosis, and high-risk patients with high *RAD54B* expression may be more suitable for immunotherapy.

## 1. Introduction

Liver cancer is a malignant tumor with a very poor prognosis. The mortality rate ranks third among all malignant tumors, accounting for approximately 8.3% of the deaths from malignant tumors [1]. HCC is the most common pathological type of primary liver cancer [2]. The survival rate of patients with extrahepatic metastasis is not ideal [3]. Lymph node is a common metastasis site of HCC, second only to the lung. It has been reported that approximately 10.3% of patients with HCC after radical resection will have lymph node metastasis (LNM) [4]. Through the lymphatic system, secondary tumor recurrence and extrahepatic metastasis will cause patients to lose the opportunity for radical surgery, which will seriously affect the prognosis of patients.

Our previous works has identified several effective biomarkers to help predict the occurrence of HCC LNM, such as *HIF-1a*, *VEGFA*, and *MMP-2*. Subsequently, some scholars also reported the effectiveness of these markers in LNM event indications of other cancer types [5–7]. Some identified biomarkers and sensitive imaging examinations can identify the existing LNM to a certain extent, but there is still a lack of systematic research on the prognosis of HCC patients with LNM. As the effectiveness of immunotherapy for liver cancer has been verified, the treatment of liver cancer tends to be diversified [8]. Therefore, more detailed biomarkers are urgently needed to guide the formulation of individualized treatment plans for liver cancer patients to further improve the prognosis.

At present, based on abundant high-throughput sequencing data, specific gene expression signatures can define the high or low risk of disease events at the molecular level. Through the identification of gene expression characteristics, several studies have reported effective gene signatures to identify disease subtypes and risk groups. Garg et al. identified a signature containing 121 metastasis-related genes based on RNA-seq data from 204 patients with primary melanoma in clinical trials. The signature was confirmed to be closely related to PFS and OS in 175 independent LNM cohorts, and the signature score was negatively correlated with immune cell infiltration [9]. Bagaev et al. reported on the establishment of a model for interpreting the tumor microenvironment (TME) by 29 characteristic gene signatures and analyzed the prognostic correlation of TME subtypes from the perspective of pancancer which could be used as a clinical indicator for predicting tumor immune response [10]. Thorsson et al. used the marker gene signature of immune cells to identify the immune subtypes of pancancer and constructed cascade networks of mutation-TF-immune subtypes based on the master regulator of each immune subtype to describe the cellular and molecular interactions involved in the tumor immune response [11]. However, there is still a lack of characteristic gene signatures that can accurately identify high-risk patients in the HCC LNM population. We speculate that there is abnormal expression of a set of characteristic gene signatures in tumor samples of HCC patients with LNM, and the prognosis-related genes contained in this gene cluster can effectively predict the prognosis of HCC LNM population.

**Figure 1:**
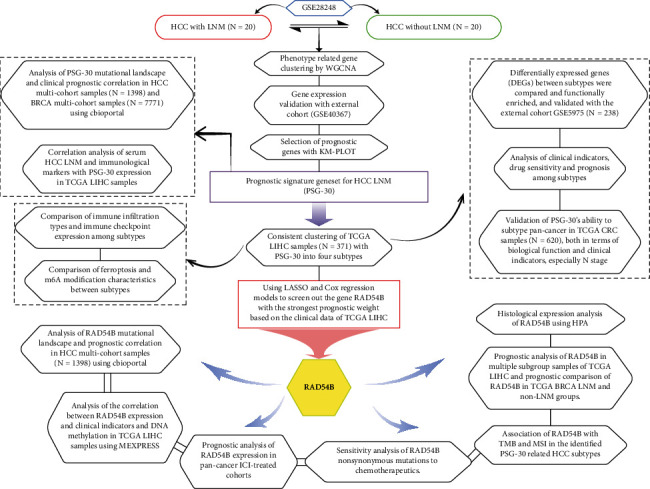
Flowchart of study design.

The study followed the steps shown in the flowchart in Figure 1 for analysis and validation. We screened the gene signature cluster (PSG-30) of HCC LNM with high prognostic correlation based on the microarray data of 20 pairs of HCC samples with and without LNM, the public pancancer transcriptome data, and the corresponding clinical data. Using this gene signature cluster, we first analyzed the differences in biological phenotype, clinical information, drug sensitivity, and immune infiltration/response among tumor samples of HCC LNM patients with different prognostic risks. M6A modification and ferroptosis activation were also analyzed in the identified subtypes, as they are broadly implicated in tumor progression, including LNM [12–15]. Secondly, the prognostic risk model was constructed, and the gene with the largest influence weight were selected from the model to comprehensively analyze its mutation landscape, clinical correlation, and histological expression. We aim to comprehensively interpret the LNM of HCC in order to provide clues for further prognosis prediction and treatment intervention.

## 2. Methods

### 2.1. Data Sources

*Data Sources from Gene Expression Omnibus (GEO,* http://www.ncbi.nlm.nih.gov/geo)

- GSE28248: cDNA microarray data of HCC tumor tissue (*N* = 40) and adjacent normal tissue samples (*N* = 40). The source patients were matched according to the history of LNM, and patients with significant statistical differences in other clinicopathological indicators were excluded. In this study, the total samples were divided into tumor tissues of HCC with LNM (*N* = 20), tumor tissues of HCC without LNM (*N* = 20), peritumoral tissues of HCC with LNM (*N* = 20), and peritumoral tissues of HCC without LNM (*N* = 20) for analysis
- GSE40367: cDNA microarray data from OCT frozen specimens (*N* = 61) of primary and metastatic liver and colon cancer. Source patients were classified according to tumor type and history of primary or metastases. In this study, 10 HCC samples without metastasis and 8 HCC/CC samples with LNM were selected for analysis
- GSE5975: oligonucleotide microarray data of HBV-positive HCC patient tumor tissue (*N* = 238). Source patients were classified based on EpCAM expression and HBV history. All 238 HCC samples were included in this study for subtype verification

*Data Sources from TCGA (* https://portal.gdc.cancer.gov)

- TCGA LIHC: sequencing dataset and corresponding clinical information of 371 HCC samples. Cases with complete prognostic information were included in further survival analysis
- TCGA CRC: sequencing dataset of all 620 colorectal cancer (CRC) samples and corresponding clinical sample information
- TCGA BRCA: sequencing dataset of all 1097 breast cancer (BRCA) samples and corresponding clinical sample information. According to the clear history of LNM, 550 samples with LNM and 514 samples without LNM were screened for analysis
- TCGA CHOL: sequencing dataset and corresponding clinical information of all 45 cholangiocarcinoma (CHOL) samples. According to the clear history of LNM, 5 samples with LNM and 26 samples without LNM were screened for analysis

*Data Sources from cBioPortal Platform (* http://www.cbioportal.org/) [16]

A total of 1026 samples from six independent HCC cohorts were used to analyze the PSG-30 gene cluster and *RAD54B* mutational landscape, survival prognosis, and distribution of clinical information. A total of 7771 samples from seventeen independent BRCA cohorts were used to analyze the mutational landscape and clinical information distribution of the PSG-30 gene cluster. Fifty-one samples from CHOL (TCGA, Firehose Legacy) were used to analyze the mutational landscape, survival prognosis, and clinical information distribution of *RAD54B*.

The transcription data related to drug sensitivity came from the Genomics of Drug Sensitivity in Cancer (GDSC, https://www.cancerrxgene.org/) database [17]. And the tissue and single-cell level expression profile data were derived from the Human Protein Atlas (HPA) https://www.proteinatlas.org/) [18].

### 2.2. Data Processing

For the unnormalized RNA-seq dataset, the original expression of each gene was log2 processed uniformly. In the microarray dataset from the GEO database, if one gene was mapped to multiple probes, the average value was taken as the expression level of the gene. The probe entries that failed to map to any gene ID or mapped to multiple gene IDs were removed. Finally, the probe IDs were converted into gene symbols according to the annotation of the corresponding microarray platform.

### 2.3. WGCNA Analysis

First, we calculated the median absolute deviation (MAD) of each gene by using the gene expression profile, eliminated the top 50% of the genes with the smallest MAD, removed the outlier genes and samples by using the gsg (goodSamplesGenes) method, and further constructed the scale-free coexpression network by using the “WGCNA” R package. Specifically, the Pearson correlation matrix and average linkage method were first used for paired genes, and the power function a|mn  = |C|mn^*β* (c | mn = Pearson correlation between gene m and gene n; a | mn = adjacency between gene m and gene n) was then used for weighted adjacency matrix construction. *β* is a soft threshold parameter that can emphasize the strong correlation between genes and punish the weak correlation. By selecting the power of 6, the adjacency can be transformed into a topological overlap matrix (TOM), which can measure the network connectivity of a gene. To classify genes with similar expression profiles into modules, average linkage hierarchical clustering was carried out according to the dissimilarity measure based on TOM. The minimum size (gene group) of the gene dendrogram was 30, and the sensitivity was set as 3. By calculating the difference in module characteristic genes, the cutting line of the module dendrogram was selected, and the modules with a distance less than 0.25 were merged. Three coexpression gene modules were finally obtained.

### 2.4. Subtype Grouping

Subtype grouping was based on RNA sequencing data and corresponding clinical information of 371 HCC tissues in TCGA dataset. Setting parameters were as follows: the maximum number of clusters was 6, 80% of the total samples were extracted 100 times, clusterAlg = ^“^HC^”^, and innerlinkage = ^“^ward.D2^”^. The consistency analysis was carried out by using the R package “ConsensusClusterPlus” (v1.54.0), and the clustering heatmap was generated using the “pheatmap” (v1.0.12) R package. The gene expression heatmap was drawn by the R packages “survival” and “surviviner,” and the genes with variances above 0.1 were retained.

### 2.5. Differential Expression Analysis and Functional Enrichment

Differential gene expression was analyzed by the “Limma” R package (version: 3.40.2). To correct the false-positive results, we further analyzed the adjusted *P* value based on TCGA or GTEX data. The differentially expressed genes were screened with adjusted *P* < 0.05 and an absolute value of log2 (fold change) > 1. Gene expression diversity dominates the changes in biological function, and enrichment analysis can link differentially expressed genes (DEGs) with related biological function items. Therefore, Gene Ontology (GO) enrichment analysis was used to annotate DEGs from three categories: molecular function (MF), biological pathway (BP), and cellular component (CC). KEGG enrichment analysis is a tool for advanced functional annotation of the genome. The “ClusterProfiler” R package was applied to analyze the correlations between DEGs and GO functions/KEGG pathways.

### 2.6. Correlation Analysis of M6A and Ferroptosis

The m6A-related genes were derived from the research by Li et al. on the molecular characterization and clinical significance of m6A modulators across thirty-three cancer types [19]. Ferroptosis-related genes were derived from the systematic analysis of Liu et al. of the abnormalities and functions of ferroptosis in cancer [20]. Correlation analysis and visualization were achieved through the “ggplot2” and “pheatmap” R packages.

### 2.7. Kaplan-Meier Survival and Drug Sensitivity Analysis

The Kaplan-Meier survival curves were based on RNA sequencing data and corresponding clinical information, which were analyzed and visualized by the “survival” and “surviviner” R packages.

Drug sensitivity analysis was based on the GDSC dataset, the chemotherapeutic response of each sample was predicted by the R package “pRRophetic,” and the IC50 value was further evaluated by the ridge regression method.

### 2.8. Immune Cell Infiltration Analysis

We used the “immunedeconv” R package for immune cell infiltration assessment, which contains six algorithms, including MCPcounter, CIBERSORT, quanTIseq, xCell, EPIC, and TIMER. The immune scoring results are displayed visually through “ggplot2” and “pheatmap” package.

### 2.9. Screening of Prognostic Factors and Establishment of Risk Model

First, the log rank test was used to detect the prognostic differences between groups compared by KM survival analysis, and the “timeroc” package was used to compare the prediction accuracy and risk score of target genes. Then, the R package “glmnet” was used to screen variables by the least absolute shrinkage and selection operator (LASSO) regression algorithm, and 10× cross-validation was applied. The selected prognostic risk factors were included in univariate and multivariate Cox regression analyses, and the *P* value, HR, and 95% CI of variables were visualized by the “forestplot” package. Furthermore, a nomogram with the chosen independent risk factors was drawn by the R package “rms” to evaluate the prognostic risk of patients at 1, 3, and 5 years.

## 3. Results

### 3.1. Identifying Prognostic Gene Signature for HCC LNM

**Figure 2:**
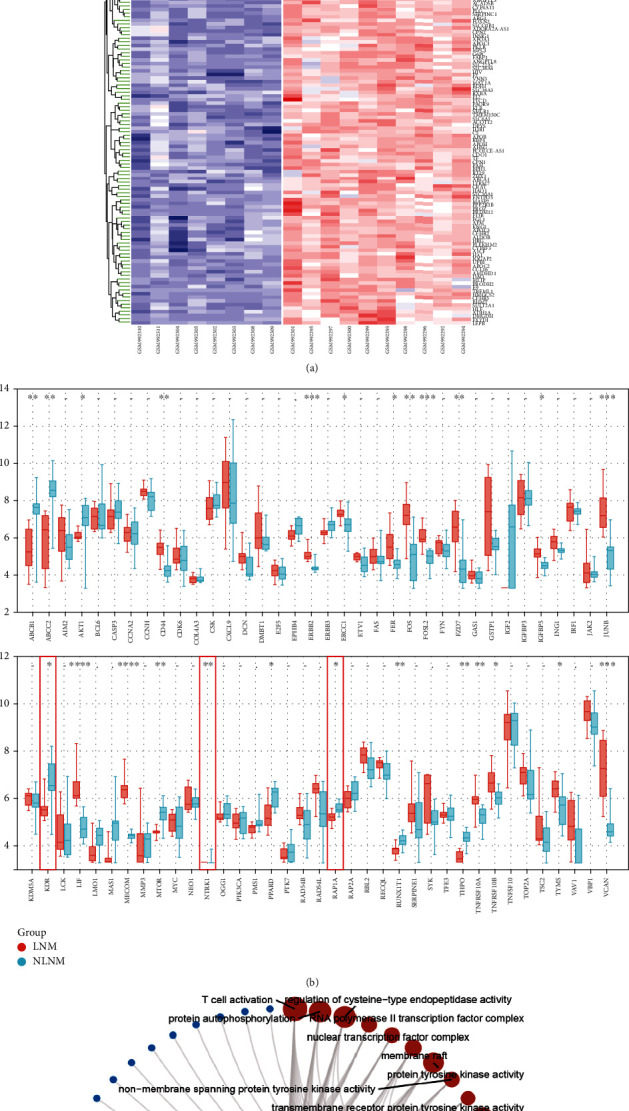
Verification and screening of genes from “blue module.” (a) In the validation set GSE40367 of the GEO database, LNM (*N* = 8) and non LNM (*N* = 10) samples of liver cancer were selected. The significant differences of gene expression between the two groups were compared by Limma test and displayed by heatmap. (b) The expression patterns of genes belonging to the blue module in the above paired groups. (c) GO and KEGG enrichment analysis of the blue module genes. The top 3 terms of each enrichment classification are highlighted in red, and the number of enriched genes is distinguished by node size.

We first analyzed the cDNA microarray data derived from GSE28248, which contained tumor and adjacent normal samples from 20 pairs of matched HCC patients with and without LNM. In this study, the threshold is selected as *β* = 6 according to the scale-free topology criterion, which is the lowest power of the scale-free topology fitting index of 0.87, and the corresponding mean connectivity is 2.6 (Supplementary Fig. 1A, B). Then, three coexpressed gene modules were obtained by WGCNA clustering and distinguished by turquoise, blue, and gray colors (Supplementary Fig. 1C, D). The clustering heatmap drawn according to the adjacency relationship shows the independence between the expression patterns distinguished by modules (Supplementary Fig. 2A). The correlation analysis between the blue module and clinical phenotype showed that there was a significant correlation between the blue module and HCC LNM (*P* = 2.9e − 6, *r* = 0.44) (Supplementary Fig. 2B, C). The coexpression relationship of 73 genes belonging to the blue module in the TCGA-LIHC sample (*N* = 424) is shown in Supplementary Fig. 2D. Then, liver cancer samples with (*N* = 8) and without LNM (*N* = 10) were selected in the GSE40367 dataset, and the heatmap for intergroup differences is shown in Figure 2(a). To refine the HCC LNM-related modules, we further aligned 73 genes belonging to the blue module in paired samples from GSE40367 (Figure 2(b)). *KDR*, *NTRK1*, and *RAP1A* genes that were significantly differentially expressed but in opposite trends between groups with and without LNM in the GSE28248 and GSE40367 datasets were excluded.  Figure 2(c) also displays the top three entries of the enrichment results of GO (BP, biological process; CC, cellular component; MF, molecular function) and KEGG pathway of the genes from the blue module.

Based on the above results, the remaining 70 genes were analyzed for the prognosis of OS (*N* = 364) and RFS (*N* = 316) through the KM plot database, and 40 genes that had no significant effect on OS and RFS of HCC were excluded. The rest 30 genes were included in the prognostic signature gene set and defined as PSG-30 for subsequent analysis (Supplementary Fig. 9 and 10).

As a complement, we analyzed the mutational landscape of PSG-30 in LIHC multicohort samples (*N* = 1026) and found that the constituent genes of PSG-30 exhibited high-frequency gene amplification (Supplementary Fig. 11A, B). The OS and DFS of the PSG-30 mutant population were significantly reduced (Supplementary Fig. 11C, D). Limited by the number of samples with a clear history of LNM, the PSG-30 mutant population did not show a significant correlation with LNM, but the PSG-30 mutant population showed more vascular macro invasion, which is a critical risk factor for HCC LNM (Supplementary Fig. 11E, F). Further, when mRNA expression was taken into account in the LIHC cohort with the highest PSG-30 mutation rate (TCGA, Firehose Legacy), PSG-30 mutation and transcriptional activity were more closely associated with poor prognosis (Supplementary Fig. 11G-I). In order to confirm the correlation between PSG-30 and LNM in pancancer, PSG-30 was also tested in BRCA multicohort samples (*N* = 7771) (Supplementary Fig. 11J, K). The results showed that PSG-30 mutation was not only related to the poor prognosis of BRCA (figure not shown). More importantly, the detection rate of positive lymph nodes in BRCA population with PSG-30 mutation is significantly higher than that in unmutated population and was more common in those with multiple positive lymph nodes (Supplementary Fig. 11L, M).

### 3.2. Consistency Clustering of HCC Samples Based on PSG-30

**Figure 3:**
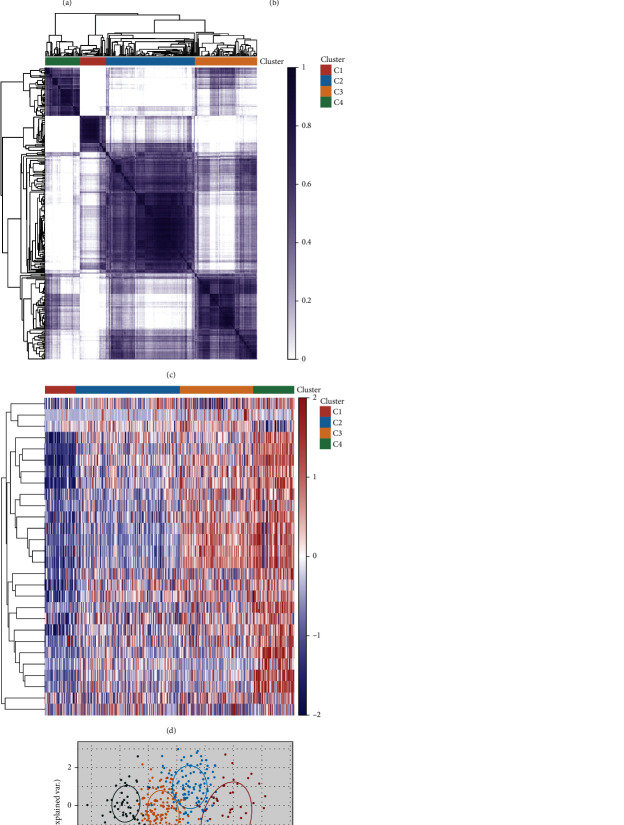
Subtype classification based on PSG-30. (a and b) Cumulative distribution function (CDF) for consensus clustering (left) and relative change in area under CDF curve for *k* = 2 − 6 (right). (c) Heatmap of consistent clustering results of 371 LIHC samples with *k* = 4. (d) Heatmap of LNM-related gene expression in the 4 subgroups (blue represents low gene expression, while red represents high gene expression). (e) The PCA diagram shows the sample separation of the four subtypes.

By comprehensively analyzing the relative change in area under the cumulative distribution function (CDF), *k* = 4 was selected as the optimum, so four subtype groups of TCGA LIHC samples (*N* = 371) were determined by the consistent clustering method (Figures 3(a)–3(c)). A cluster heatmap showed that there was a significant difference in the expression of the PSG-30-related genes among the groups, and the characteristic expression of PSG-30 showed an upward trend from cluster 1 to cluster 4 (Figure 3(d)). The PCA diagram visually distinguished the differences in gene expression patterns among subtypes (Figure 3(e)). Subtype 4 is the subtype with the highest expression of PSG-30 signature. Compared to baseline subtype 1, subtype 4 emphasizes cancer cell-stromal interactions, cell cycle dysregulation, and immune dysregulation (Supplementary Fig. 3).

A total of 238 HCC samples from the GSE5975 dataset were further collected, and the subtype classification was verified by PSG-30 (Supplementary Fig. 4A). After that, the samples were divided into 4 subtype groups, and group 2 was taken as the baseline because the number of group 1 samples was too small (Supplementary Fig. 4B). Compared with group 2, group 4 accumulated abnormal activity of cancer cell-matrix interactions and cell cycle dysregulation (Supplementary Fig. 4C-E). It is worth noting that the enrichment of immune-related terms decreased compared with the results from TCGA LIHC dataset. We speculate that the cases contained in GSE5975 are HBV positive, and the original background of tumor tissue carries interference factors of immune tolerance.

### 3.3. Multidimensional Comparison between HCC Subgroups Distinguished by PSG-30

**Figure 4:**
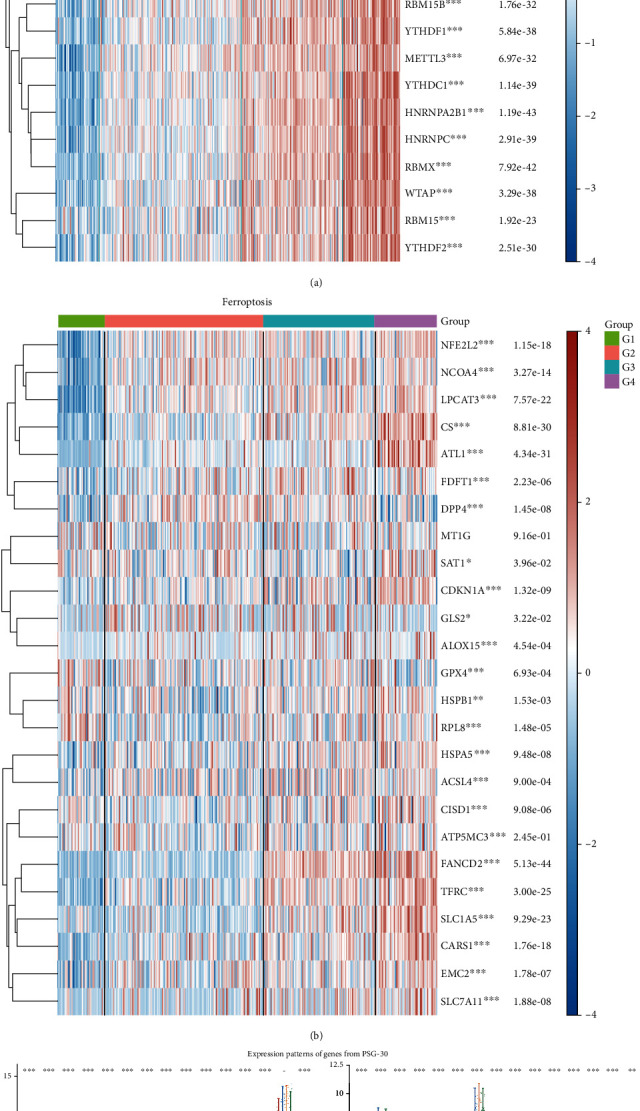
Correlation analysis between functional gene expression and identified subgroups. (a) Correlation analysis between subgroups and m6A modification: the horizontal axis represents different subtype groups, and the vertical axis represents m6A related gene expression. (b) Correlation analysis between subgroups and ferroptosis: the horizontal axis represents different subtype groups, and the vertical axis represents ferroptosis related gene expression. Different colors represent the expression trend in each subgroup. (c) Differential expression of PSG-30 genes between the four subtypes (^∗^*P* < 0.05, ^∗∗^*P* < 0.01, and ^∗∗∗^*P* < 0.001).

Through the correlation analysis between subtype groups and m6A regulatory factor expression, we found that there was a significant positive correlation between m6A modification activity and PSG-30 expression level (Figure 4(a)). Likewise, we found that ferroptosis-related genes were significantly activated in the subtypes with high PSG-30 expression (Figure 4(b)). The distribution plot showed the expression level and trend of each gene contained in PSG-30 in the four subtypes (Figure 4(c)), which indicates that the characteristics of PSG-30 may contribute to the malignant phenotype of tumor cells through m6A modification and ferroptosis activation.

**Figure 5:**
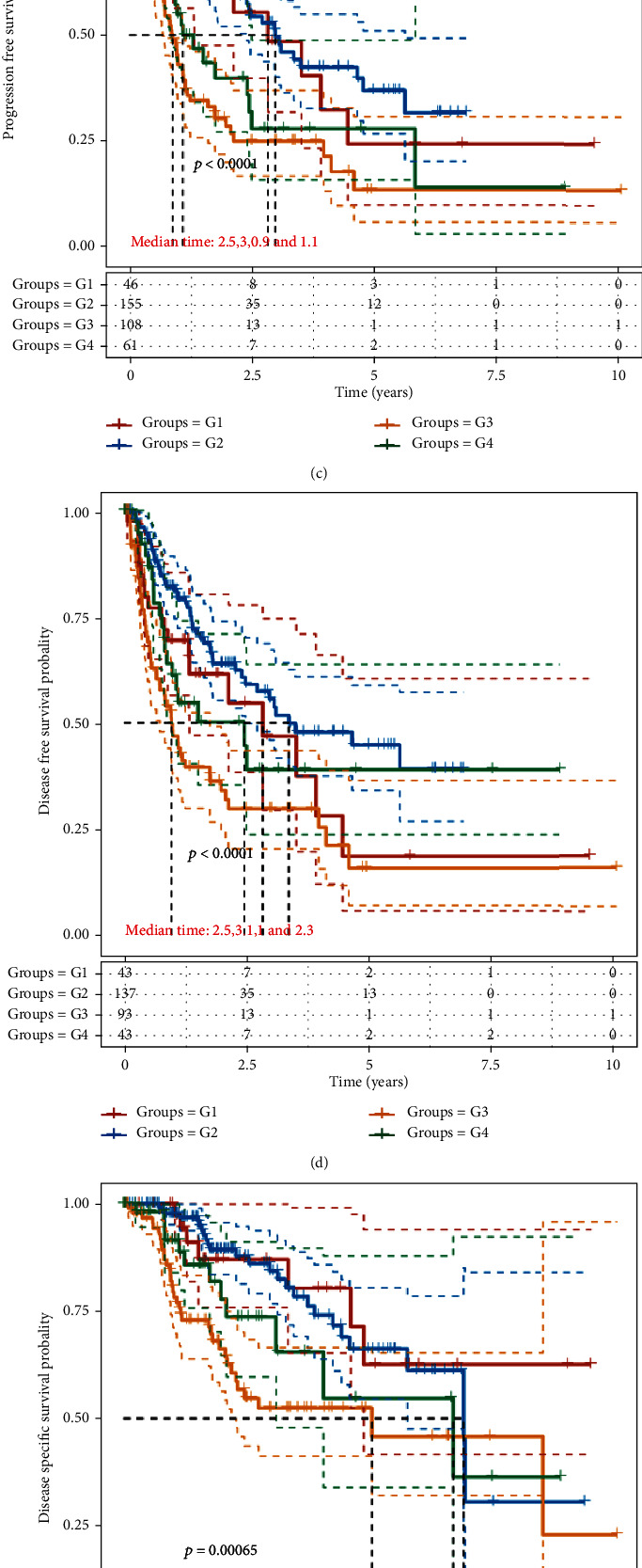
Clinicopathological analysis of identified HCC subtypes. (a) The distribution of clinical characteristics in samples of different subtype groups, in which the horizontal axis represents different groups, the vertical axis represents the percentage of clinical information contained in corresponding grouped samples, and different colors represent different clinical information. The above table represents the distribution of a clinical feature in any two groups. The marked value is -log10 (*P* value), and the ∗ represents significant difference between two groups (^∗^*P* < 0.05). (b–e) Kaplan-Meier analysis of prognosis of subtype groups in TCGA LIHC data set. Different groups were tested by log-rank method and *P* < 0.05 depict statistically significant difference between groups ((b) OS, *P* = 0.0078; (c) PFS, *P* < 0.0001; (d) DFS, *P* < 0.0001; and (e) DSS, *P* = 0.00065). Mean time represents the median survival time in years. (f) Box diagram of IC50 score distribution of sorafenib in different subtype groups, in which the horizontal axis represents samples of different groups (different colors), and the vertical axis represents IC50 score distribution (Kruskal Wallis test, ^∗∗∗∗^*P* < 0.0001; ns: not significant). (g) A Sankey diagram shows the overlapping areas and proportions of PSG-30 genes and known functional cluster genes.

In addition, we compared the clinical information among the subtype groups. The results showed that subtypes with high PSG-30 expression were related to more advanced T stage, TNM stage, and pathological stage. Limited by the small number of LNM cases included in TCGA LIHC samples (*N* = 4), the N stage showed a sufficient significant difference. However, it is noteworthy that samples with LNM were concentrated in PSG-30 high expression subtypes (Figure 5(a)).

Survival analysis highlighted significant prognostic differences between subtype groups, which were manifested in OS (*P* = 0.0078), PFS (*P* < 0.0001), DFS (*P* < 0.0001), and DSS (*P* = 0.00065) (Figures 5(b)–5(e)). The above results indicated that high PSG-30 expression was significantly associated with worse prognosis. The efficacy of the targeted drug sorafenib in advanced liver cancer has been recognized. Single drug application prolongs the time to progression (TTP) and improves the OS of patients [21]. Based on the Genomics of Drug Sensitivity in Cancer (GDSC) and TCGA LIHC transcriptome data, IC50 predictions were made for drug sensitivity to sorafenib for each identified subtype (Figure 5(f)). We found that the PSG-30 high expression group was more sensitive to sorafenib, suggesting that the drug may have better efficacy in HCC patients with LNM.

As a complement, we performed a cross-over analysis between PSG-30 and the gene sets associated with common tumor malignancies and displayed in the Sankey diagram. The concordance of PSG-30 with energy metabolism, DNA damage repair, immunity, and EMT signals was 6.7%, 10%, 23.3%, and 50%, respectively (Figure 5(g)). This implies that multiple malignant phenotypic modules synergistically promote the lymph node metastatic behavior of HCC, in which disturbances of EMT and immune-related functions are major contributing factors.

From a pancancer perspective, PSG-30 can also distinguish subtypes of CRC with high lymph node metastasis potential. Using PSG-30 to divide TCGA CRC samples (*N* = 620) into four subtype groups, we noticed the emergence of abnormally active “plates” of PSG-30 expression in group 4 (Supplementary Fig. 5A). Enrichment analysis of differentially expressed genes between groups 1 and 4 revealed that significantly disrupted cell adhesion and T cell function occurred in group 4 (Supplementary Fig. 5B-D). We further demonstrated a highly activated state of myeloid cells in group 4 by immune infiltration analysis based on the xCell algorithm (Supplementary Fig. 5E). The results of clinical data analysis indicated that the cases in group 4 had higher T stage and TNM stage. And more importantly, the number of cases with LNM in group 4 increased significantly and mainly clustered in the N2 stage (Supplementary Fig. 5F).

**Figure 6:**
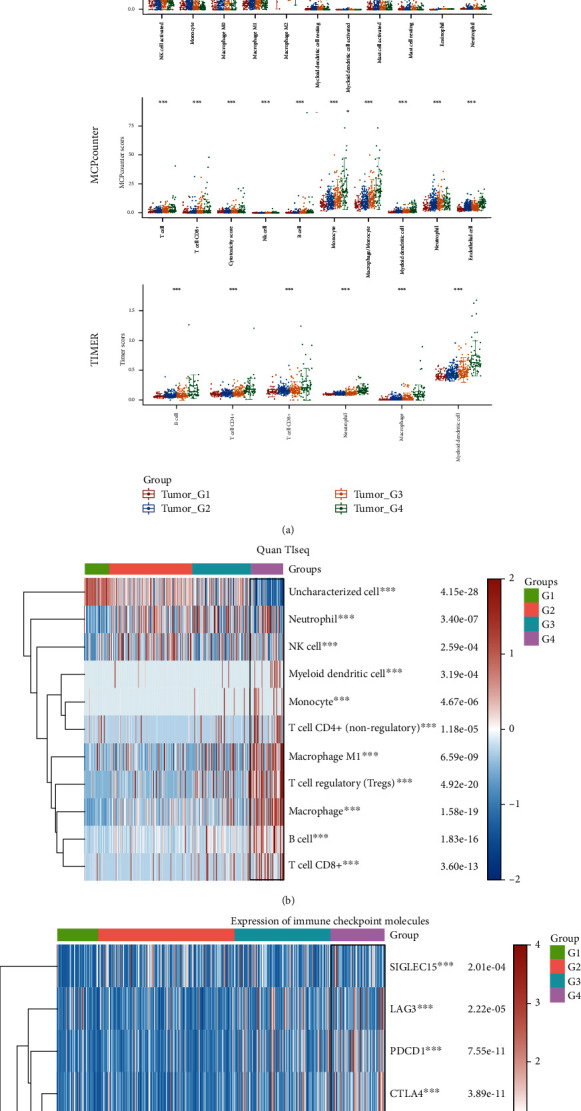
Comprehensive immune infiltration score and immune checkpoint-related molecular expression analysis in the identified subgroups. (a) A box plot shows the distribution of immune score from xCell, MCPcounter, and TIMER in four subtypes related to HCC LNM, in which the horizontal axis represents the type of immune cells and the vertical axis represents the distribution trend of the immune score between subtypes. (b) The heatmap shows the distribution of tumor infiltrating immune cells between subtype groups evaluated by quanTIseq method, in which different colors highlight the differences in the composition of infiltrating immune cells between subtypes. (c) A heatmap displays the expression and distribution of immune checkpoint related genes among samples of the four subtypes. ^∗^*P* < 0.05, ^∗∗^*P* < 0.01, and ^∗∗∗^*P* < 0.001, Kruskal-Wallis test.

We also assessed the immune infiltration of the identified subtypes of HCC based on four algorithms, CIBERSORT, MCPcounter, TIMER, and quanTIseq. The results showed that the degree of infiltration of myeloid cells, especially macrophages, was closely related to the high-risk group of LNM (Figures 6(a) and 6(b)). Immune checkpoint molecules that mediate immune suppression and immune escape were also overexpressed in high-risk subtypes (Figure 6(c)).

### 3.4. Screening of Prognostic Markers in HCC Patients with LNM

First, we selected the subtype 4 with the most significant characteristics of PSG-30 as the training set (*N* = 61) and screened the genes contained in PSG-30 by the LASSO regression model [22]. Seven genes were included in the optimal OS prediction model: lambda.Min = 0.0658, risk score = (−0.1021)∗*CD*44 + (0.0541)∗*FZD*7 + (0.0613)∗*LIF* + (−0.0951)∗*BCL*6 + (0.2979)∗*CCNA*2 + (1.2388)∗*RAD*54*B* + (0.4193)∗*RECQL* (Supplementary Fig. 6A-E). The correlation analysis of the risk score and immune score was carried out by using TIMER and quanTIseq algorithms. The results showed that the model risk score was significantly negatively correlated with NK cell and CD8+ T cell infiltration (*P* = 0.001, *P* = 0.028) (Supplementary Fig. 6F, G). Similarly, we build the optimal DSS prediction model: lambda.Min = 0.0827, risk score = (0.0206)∗*MYC* + (1.4093)∗*RAD*54*B* (Supplementary Fig. 7A-E). Both the OS- and DSS-based risk prediction models described above showed the great weighting of *RAD*54*B*.

*PD-L1* (*CD*274) expression level, TMB, and MSI are putative indicators to predict the efficacy of immunotherapy [23]. Figure 6(c) revealed significantly elevated PD-L1 expression in samples from subtype 4. We then analyzed the correlation between the expression level of *RAD54B* and TMB and MSI in the identified HCC subtypes. It was obvious that the expression of *RAD54B* in subtype 4 was significantly positively correlated with TMB and MSI but not in other subtypes (Supplementary Fig. 7F-G).

**Figure 7:**
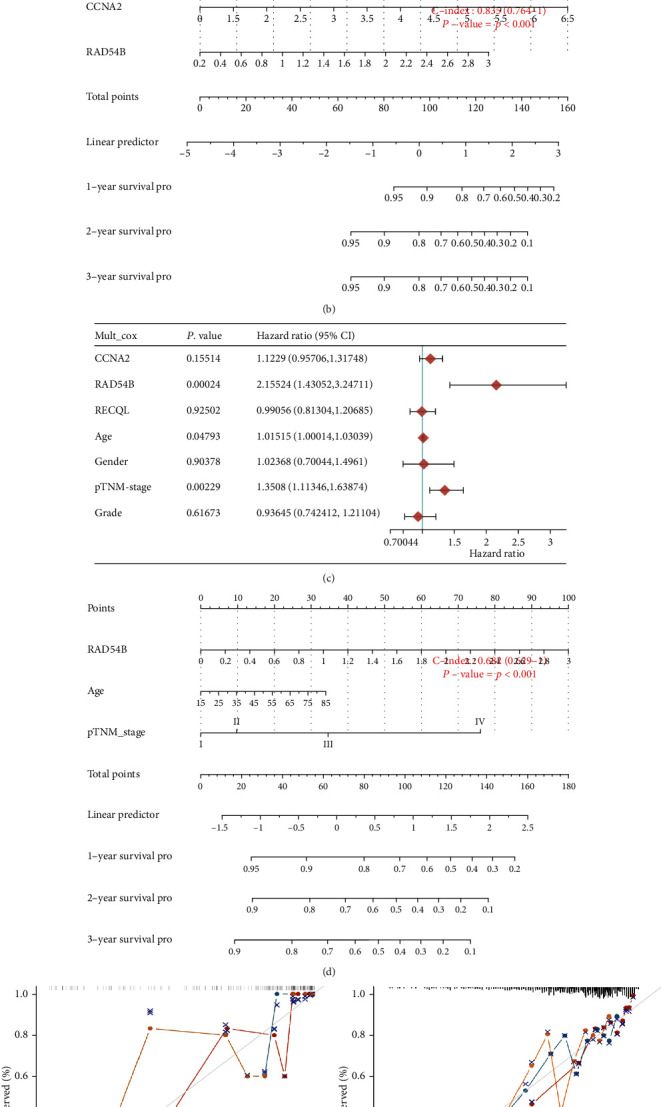
Construction and validation of nomograms for risk prediction of OS in HCC. (a and c) The risk factors *CCN2*, *RAD54B*, and *RECQL* from the established LASSO regression model for OS and the recorded clinical factors age, gender, pTNM stage, and pathological grade were included in multivariate logistic regression analysis to determine the independent risk factors for OS in the identified HCC subtype 4 cases (a) and total TCGA LIHC cases (c). (b and d) Construction of nomogram predicting 1-, 2-, and 3-year OS for patients from the identified HCC subtype 4 (*P* < 0.001, C-index: 0.835, 95% CI: 0.764-1) (b) and total TCGA LIHC (*P* < 0.001, C-index: 0.682, 95% CI: 0.629-1) (d). (e and f) Calibration curve of nomogram for subtype 4 (e) and total TCGA LIHC (f).

Additionally, we included the top three risk factors from the LASSO regression model for OS constructed in subtype 4 above, as well as clinical indicators, into the multivariate regression analysis of the identified subtype 4 and total TCGA LIHC cases (Figures 7(a) and 7(c)). In subtype 4 cases, *RAD54B* (*P* = 0.00342, HR: 4.78, 95% CI: 1.68-13.63) and *CCNA2* (*P* = 0.032, HR: 2.34, 95% CI: 1.07-5.09), as independent risks for OS factors, were incorporated into risk models and plotted as nomograms to help predict 1-, 2-, and 3-year survival (*P* < 0.001, C − index = 0.835, 95% CI: 0.764-1) (Figure 7(b)). The calibration curve in Figure 7(e) is not ideal due to the lack of samples. However, in total TCGA LIHC cases, *RAD54B* also appeared to be the most weighted independent risk factor (*P* = 0.00024, HR: 2.16, 95% CI: 1.43-3.24). The nomogram composed of *RAD54B*, age, and pTNM stage had better predictive performance and reliability (Figures 7(d) and 7(f)).

### 3.5. Genomic Landscape and Clinical Correlations of RAD54B in HCC

**Figure 8:**
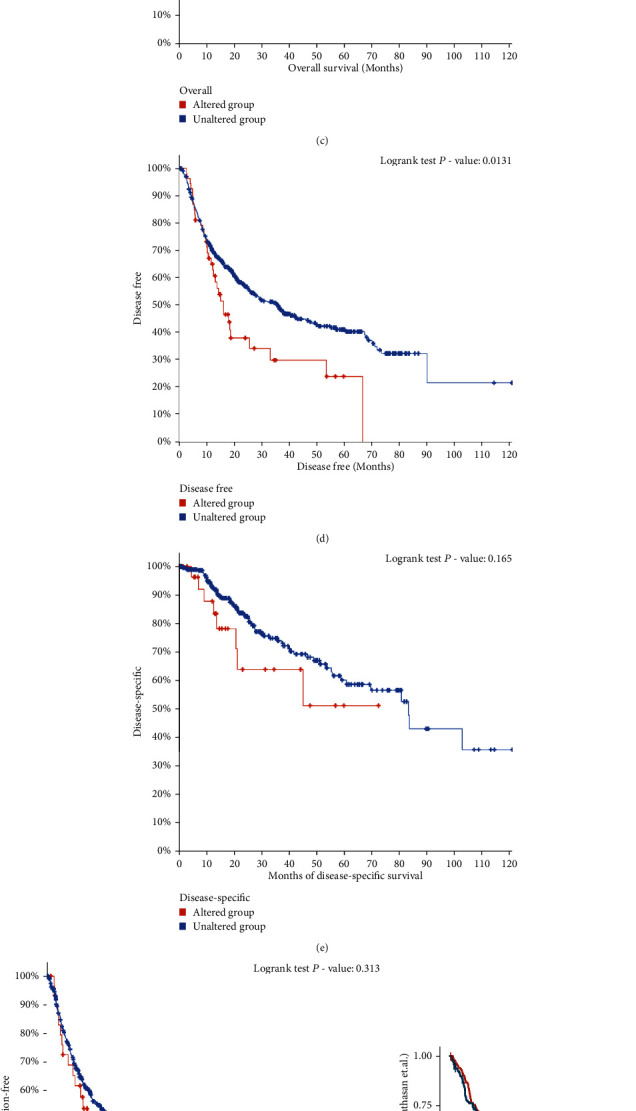
The mutation view of *RAD54B* and its clinical relevance. (a) The mutation landscape of *RAD54B* in 1026 samples of 1022 patients in 6 HCC cohorts and the comparison of mutation frequency of *RAD54B* in each cohort from cBioPortal platform. (b) The correlation between the expression of *RAD54B* in HCC and the clinical characteristics (upper) or DNA methylation level (below) was analyzed by MEXPRESS tool. (c–f) The prognosis of the *RAD54B* mutated and unmutated groups in 6 cohorts of patients was analyzed on the cBioPortal platform ((c) OS, *P* = 1.310e − 4; (d) DFS, *P* = 0.0131; (e) DSS, *P* = 0.165; and (f) PFS, *P* = 0.313). (g and h) The effects of *RAD54B* expression on the OS of patients was analyzed in the ICI treatment cohorts for bladder cancer (*P* = 0.007, HR = 0.7, 95% CI: 0.54-0.91) and melanoma (*P* = 0.015, HR = 0.34, 95% CI: 0.13-0.89). (i) Drug sensitivity analysis based on the GDSC, the horizontal axis shows the group (wild type and mutant), and the vertical axis shows the given drug and the corresponding ln(predicted IC50) value.

To comprehensively evaluate the mutation landscape of *RAD54B* in HCC patients, we applied the cBioPortal platform to analyze 6 independent HCC cohorts. *RAD54B* had genomic changes in 7% of the total population, in which gene amplification events accounted for the majority, especially in the cohort (TCGA, Firehose Legacy) (Figure 8(a)). Further analysis of TCGA LIHC data using the MEXPRESS platform showed that *RAD54B* expression in tumor tissue was inversely correlated with age at initial pathologic diagnosis and OS. Its expression also showed a significant correlation with Child-Pugh grade, histological grade, new neoplasm event type, relative family cancer history, and race. From Figure 8(b), we can also infer that high expression of *RAD54B* is associated with copy number variation (CNV). Demethylation appeared to simultaneously promoted the overexpression of *RAD54B*, especially at the two methylation sites cg26323655 (*r* = −0.291, *P* < 0.001) and cg25305774 (*r* = −0.239, *P* < 0.001).

Based on the cBioPortal platform, we conducted a prognosis test in 1022 cases out of 6 independent cohorts, and the prognosis of the *RAD54B*-altered group was significantly worse than that of the unaltered group (Figures 8(c)–8(f)). Interestingly, high *RAD54B* expression was significantly associated with better OS in the ICI treatment cohorts of bladder cancer (Mariathasan et al.) and melanoma (Auslander et al.) (Figures 8(g) and 8(h)). Moreover, GDSC susceptibility analysis indicated that the *RAD54B* mutant population was highly sensitive to pyrimethamine and gefitinib. The above results show that the efficacy of ICI therapy combined with pyrimethamine or gefitinib is worth observing, especially in patients with *RAD54B* overexpression (Figure 8(i)).

**Figure 9:**
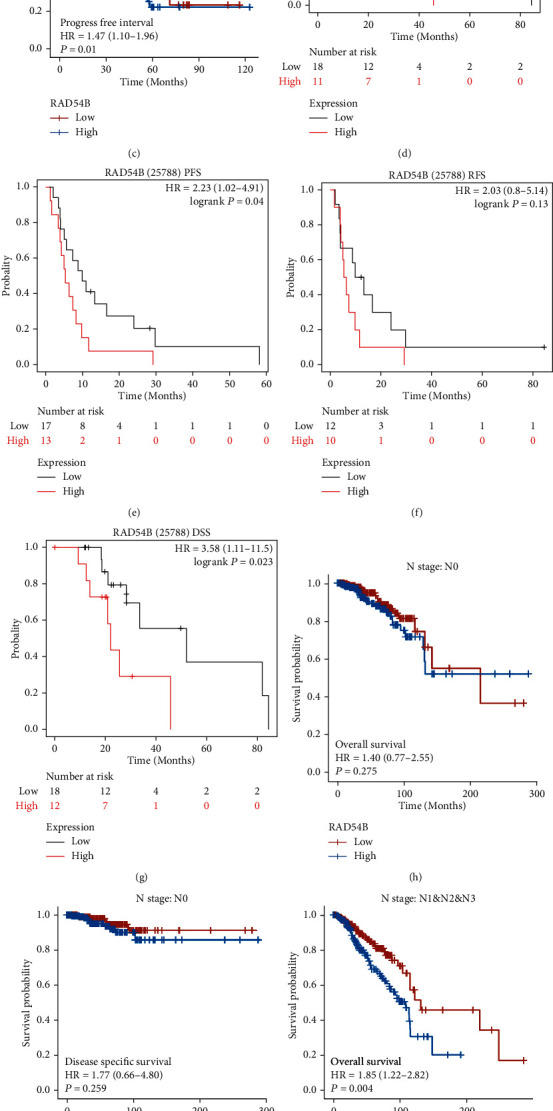
Prognostic analysis of *RAD54B* in HCC subgroup and other cancer types. (a–c) The prognosis of *RAD54B* was analyzed in the total TCGA sample (*N* = 371) ((a) OS, *P* = 0.001, HR = 1.82, 95% CI: 1.28-2.58; (b) DSS, *P* = 0.004, HR = 1.92, 95% CI: 1.23-3.01; and (c) PFS, *P* = 0.01, HR = 1.47, 95% CI: 1.10-1.96). (d–g) KM plot tool was used to analyze the effects of high and low expression of *RAD54B* on prognosis in sorafenib treatment group of HCC ((d) OS, *P* = 0.023, HR = 3.58, 95% CI: 1.11-11.50; (e) PFS, *P* = 0.04, HR = 2.23, 95% CI: 1.02-4.91; (f) RFS, *P* = 0.13; and (g) DSS, *P* = 0.023, HR = 3.58, 95% CI: 1.11-11.50). (h–k) The breast cancer samples were divided into LNM and non LNM groups, and the prognosis of *RAD54B* expression was analyzed. *RAD54B* showed significant prognostic value only in LNM group ((h) N0-OS, *P* = 0.275; (i) N0-DSS, *P* = 0.259; (j) N1&2&3-OS, *P* = 0.004, HR = 1.85, 95% CI: 1.22-2.82; and (k) N1&2&3-DSS, *P* = 0.015, HR = 1.90, 95% CI: 1.13-3.19).

As a complement, we found that compared with the prognostic differences in the TCGA LIHC dataset shown in Figures 9(a)–9(c), the prognosis of the *RAD54B* high-expressing population in the sorafenib-treated group (*N* = 30) was significantly worse (Figures 9(d)–9(g)). BRCA has the highest incidence of LNM among malignancies, and multiple identified markers of BRCA LNM are consistent with markers of HCC LNM [24–27]. Therefore, to make up for the lack of HCC LNM samples in the TCGA dataset, we compared the prognostic differences between the high and low *RAD54B* expression groups in TCGA BRCA samples with (*N* = 550) and without LNM (*N* = 514). As shown in Figures 9(j) and 9(k), in the LNM group (N stage: N1+N2+N3), the prognosis of the *RAD54B* high expression group was significantly worse than that of the *RAD54B* low expression group. However, in the non-LNM population (N stage: *N* = 0), the expression level of *RAD54B* was not significantly associated with the prognosis of patients (Figures 9(h) and 9(i)).

Regarding the possible interference of HBV present in the above-mentioned GSE5975 gene set on PSG-30-related subtype classification, we additionally analyzed the prognosis of hepatitis virus-positive samples (*N* = 153) and virus-negative samples (*N* = 169) in TCGA LIHC. The high expression of *RAD54B* in the hepatitis virus negative group (Supplementary Fig. 8A-D) showed a more significant association with poor prognosis compared to the hepatitis virus-positive group (Supplementary Fig. 8E-H).

### 3.6. Histological Analysis of RAD54B Expression

**Figure 10:**
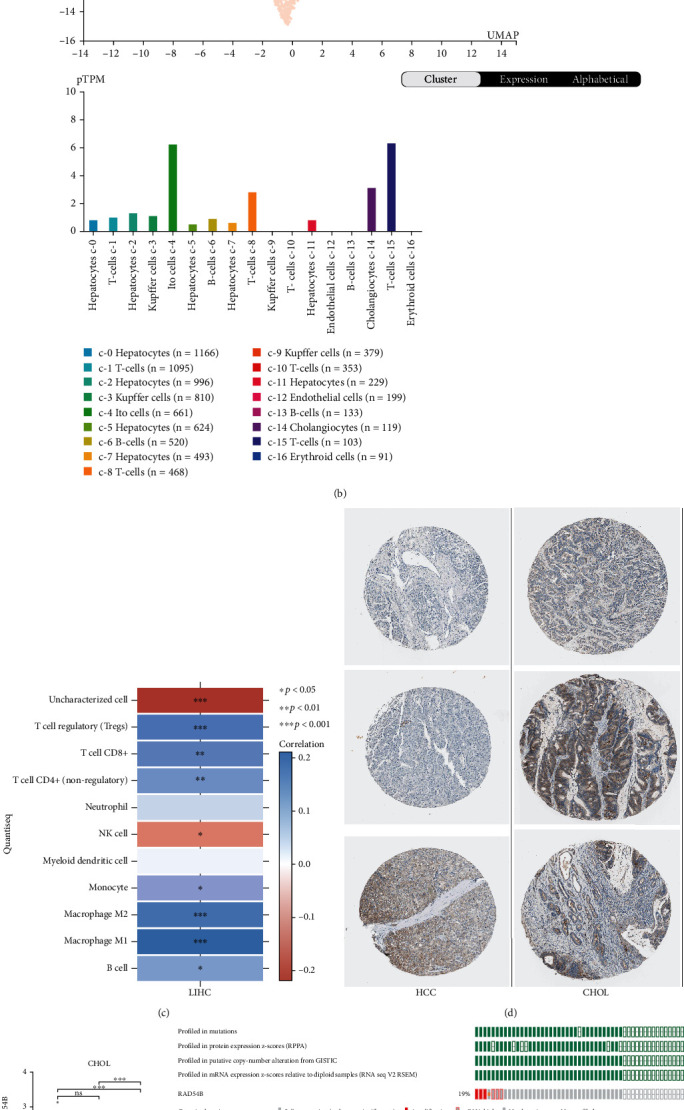
Histological overview of *RAD54B* and its potential role in cholangiocarcinoma. (a) Expression and distribution of *RAD54B* protein and RNA in different tissues and organs in HPA database. (b) Single-cell analysis of differences in *RAD54B* expression among different types of cell clusters in the liver. (c) Relationship between *RAD54B* and immune cell infiltration in HCC calculated by quanTIseq method. (d) The HPA database was used in order to determine the *RAD54B* protein expression level in HCC and CHOL via immunohistochemistry (IHC) staining, and the typical IHC images were obtained from the HPA database. (e) Comparison of *RAD54B* mRNA expression levels in subgroups N0, N1, and normal tissues in TCGA CHOL dataset. (f and g) The gene mutation and mRNA overexpression of *RAD54B* in TCGA CHOL (Firehose Legacy) samples and the correlation analysis of the altered group with LNM. (h and i) Prognostic analysis of the *RAD54B* mutation and mRNA overexpression group in the TCGA CHOL (Firehose Legacy) cohort.

We performed tissue expression analysis on the Human Protein Atlas (HPA) platform.  Figure 10(a) shows that *RAD54B* is highly expressed in lymphoid tissues at both the protein and RNA levels but is expressed at low levels in normal liver organs. Furthermore, single-cell level analysis revealed that *RAD54B* was highly expressed in Ito cells, T cells, and cholangiocytes but expressed at lower levels in normal hepatocytes (Figure 10(b)). Through the above analysis, we observed that *RAD54B* seems to have a basal high expression in the immune tissues and infiltrating immune cells of the liver. Therefore, based on the quanTIseq algorithm, the expression of *RAD54B* in TCGA LIHC samples was revealed to be significantly positively correlated with Tregs and macrophage infiltration (*P* < 0.001) (Figure 10(c)).

CHOL is another pathological type of liver cancer, which occasionally coexists with HCC. Moreover, compared with HCC, CHOL patients tend to develop LNM earlier and more frequently. Immunohistochemical results showed that the expression intensity of *RAD54B* in HCC was generally weaker than that in CHOL (Figure 10(d)).

Therefore, based on TCGA CHOL samples (*N* = 45), we found that *RAD54B* was significantly increased in CHOL compared to normal tissue. However, due to the limitation of the sample size, the expression of *RAD54B* in the CHOL LNM group (*N* = 5) was increased but not statistically significant compared with the non-LNM group (*N* = 26) (Figure 10(e)). Further, in TCGA CHOL (Firehose Legacy) dataset, 19% of the samples had genomic alterations of *RAD54B*, which were significantly associated with LNM (*P* = 0.0117) (Figures 10(f) and 10(g)). Cases with genomic alterations did not show significant prognostic differences and may require larger numbers of samples to support (Figures 10(h) and 10(i)).

## 4. Discussion

This study first established the prognosis-related gene marker PSG-30 in HCC patients with LNM, and differentiated HCC patients into subtypes with significantly different prognosis. Compared with the low-risk subtype group, the tumor tissue of the high-risk subtype group showed significantly elevated m6A methylation modification, ferroptosis activation, and drug sensitivity to sorafenib. Moreover, the infiltration of myeloid cells, especially macrophage, was significantly increased in samples from the high-risk subtype group, and the expression of corresponding immune checkpoint molecules was upregulated. Based on this, we found that *RAD54B* in the PSG-30 gene cluster was an independent risk factor for the prognosis of HCC LNM. High *RAD54B* expression was associated with worse prognosis in samples from the sorafenib-treated cohort and hepatitis virus-negative cohort. However, high expression of *RAD54B* also brings potential increased benefits of ICI treatment.

From a pancancer perspective, we selected CRC as the validation group for PSG-30 subtype grouping. The reason is that there are a sufficient number of LNM samples in the TCGA CRC dataset. Furthermore, CRC is most prone to liver metastases, and its colonization is not only related to circulating blood flow but also depends on a favorable metastatic microenvironment in the liver [28–30]. Therefore, we speculate that there is a close biological link between CRC and HCC, especially in the tumor microenvironment (TME) [31, 32]. The results showed that the high- and low-risk subtypes differentiated by PSG-30 were similar to HCC subtypes in terms of biological function enrichment differences, and their high-risk subtypes also showed the same significant macrophage infiltration as HCC. More importantly, CRC samples with LNM were significantly enriched in the high-risk subtype group.

We also selected BRCA samples for validation. The first reason is that the incidence of LNM is highest in BRCA, and the samples available for analysis are abundant. Moreover, the biomarkers of LNM for BRCA and HCC have high concordance among existing studies. We found that gene amplification of PSG-30 dominated the genetic alteration landscape of BRCA samples, and the altered group had significantly worse prognosis, LNM rate, and number of positive lymph nodes. Interestingly, *RAD54B* expression, an independent risk factor for HCC LNM, was also significantly associated with prognosis in the BRCA LNM group, but not in the LNM-free group.

We further analyzed the protein and mRNA expression of *RAD54B* at the tissue level. We found that *RAD54B* was significantly highly expressed in lymphoid tissues, and the high expression of *RAD54B* in tumor tissues with LNM may be partly due to the contribution of tumor neoplastic lymphatic vessels, which proved to be associated with LNM. Single-cell expression analysis highlighted the high baseline expression of Ito cells, T cells, and cholangiocytes in normal liver tissue. Among them, Ito cells and T cells are involved in the immune response, so we specifically analyzed the correlation between the mRNA expression of *RAD54B* and the type of immune cell infiltration in HCC. The results showed that the correlation between prominent Tregs and macrophage infiltration was more significant than that of CD4+/CD8+ T cells, and NK cell infiltration was negatively correlated. When considering cholangiocytes, we additionally analyzed the expression profile and mutational landscape of CHOL, another histological type in liver tumors. The results demonstrate a high mutation rate and tissue expression of *RAD54B* in CHOL. Notably, *RAD54B* mRNA had a trend of high expression in the LNM group of CHOL. Moreover, when high mRNA expression was considered together with gene amplification, *RAD54B* was significantly associated with CHOL LNM (*P* = 0.0345).

We noticed that the identified HCC LNM-related recognition module contained 70 genes, which were mainly related to the regulation of endopeptidase activation, T cell activation, and intercellular adhesion. Current studies have found that a variety of endopeptidases are specifically highly expressed in cancer and play a role in promoting cancer. Asparagine endopeptidase can mediate the formation of malignant tumor phenotype and adverse tumor microenvironment by acting on substrate proteins such as P53, integrin, and matrix metalloproteinase, thereby promoting the occurrence, development, and metastasis of malignant tumors [33]. Neutral endopeptidase expression in CRC cells can enhance liver metastasis of CRC by degrading the hepatoprotective methionine enkephalin [34]. Meanwhile, cells can acquire enhanced proliferation and metastatic ability by overexpressing the neural cell adhesion molecule L1 (L1-CAM), and the expression of neutral endopeptidase is a necessary condition for the acquisition of L1 characteristics of cancer cells [35]. Regulation of T cell activation also plays a dominant role in tumor metastasis, especially in the immune microenvironment of early LNM. In metastatic sentinel lymph nodes, the effects of metastatic BRCA cells on the immune system focus at an early stage on sustained T-cell immune responses, depletion of effector T cells, and enhanced Treg-mediated immunosuppression [36–38]. In addition, *γδ* T cells can promote BRCA LNM through neutrophil-mediated CD8+ T cell suppression [39]. Regulation of cell adhesion plays a cornerstone role in the formation of the tumor-associated microenvironment and tumor cell metastasis. Studies have found that intercellular adhesion molecule-1 (ICAM-1) of tumor cells plays a role in tumor progression by promoting malignant phenotype of cancer cells, activity of angiogenesis/lymphangiogenesis, and macrophage infiltration and is significantly associated with LNM [40].

As shown in the Sankey diagram shown in Figure 5(g), the screened PSG-30 gene modules overlapped with gene modules associated with tumor malignant phenotypes, especially EMT, immunity, DNA damage repair, and energy metabolism. The different weights of each overlapping module underscored the advantages of EMT and immunity in LNM of poor prognosis HCC, both of which are mainly related to the remodeling of the TME. Significantly upregulated m6A modification and ferroptosis activation in high-risk subtype tissues also suggested the occurrence of TME remodeling [41, 42]. Our studies have highlighted the correlation between macrophage infiltration and high-risk populations in HCC LNM. Tumor-associated macrophages (TAMs) are macrophages infiltrating tumor tissues, which are closely related to LNM in various tumor types [43–45]. Weichand et al. reported the relationship between inflammatory macrophages and tumor lymphangiogenesis in pancancer and confirmed the relationship between the expression of inflammasome NLRP3 in TAMs and LNM [46]. Chen et al. reported that LNMAT1-induced upregulation of CCL2 recruited TAMs and further promoted LNM in bladder cancer by secreting VEGF-C [47]. PDPN-expressing macrophages (PoMEs) have been validated to promote tumor lymphangiogenesis and LNM in BRCA [48]. Interestingly, this study found a significant positive correlation between PSG-30 and TAM/M2 macrophage markers in TCGA LIHC samples, which further corroborated the promoting effect of TAM on HCC LNM (Supplementary Fig. 12B). The significant expression correlation between PSG-30 and HCC LNM-related free mRNA screened from the microarray in our previous study also expanded the potential value of PSG-30 in clinical application (Supplementary Fig. 12A) [49].

*RAD54B* belongs to the *SWI2/SNF2* gene superfamily and is mainly involved in the homologous recombination repair process of DNA. *RAD54B* detects copy number variations, single nucleotide polymorphisms, and aberrant mRNA expression in multiple tumor types and has been shown to be closely related to tumor prognosis. In hepatoma cell lines, *RAD54B* was significantly higher expressed than in normal hepatocytes. Moreover, high expression of RAD54B protein in liver cancer tissues was significantly associated with poor prognosis. Studies have reported that *RAD54B* can promote the metastatic properties of liver cancer cells through the Wnt/*β*-catenin signaling axis. In lung adenocarcinoma, simultaneous expression of RAD54B and FEN1 proteins was also associated with late-stage LNM in patients. According to our study, gene amplification and increased transcription of *RAD54B* were significantly associated with the prognosis of HCC, which was also manifested in BRCA. The high-risk HCC subtypes identified in this study were analyzed to have higher sorafenib sensitivity and high expression of immune checkpoint molecules, suggesting that this population is more suitable for sorafenib combined with ICI therapy. However, in the above-mentioned high-risk subtype samples, while high *RAD54B* expression suggested a worse prognosis, it was significantly positively correlated with increased TMB/MSI and showed a worse prognosis in the sorafenib-treated group. This suggests that for the HCC LNM population with high expression of *RAD54B*, sorafenib may not be the first choice drug, but the status of immunotherapy is further highlighted. The results of susceptibility analysis provided gefitinib as a combination option.

To date, many advances have been made in the immunotherapy of advanced liver cancer. Patients who received immunotherapy had significantly improved outcomes compared with treatment with sorafenib alone. Atezolizumab combined with bevacizumab has become the first-line treatment for patients with unresectable or metastatic HCC [50]. Here, our study provides more nuanced evidence for immunotherapy in HCC patients with LNM.

There are some limitations of our work. First, there is a lack of sufficient HCC LNM samples to directly validate PSG-30 as well as prognostic models. Furthermore, considering that the findings were analyzed and obtained based on datasets from bioinformatics and public databases, it is necessary to further validate the recognition ability of PSG-30 and the prognostic role of *RAD54B* in a larger independent cohort. The molecular mechanisms of HCC TNM-related genes in vivo and in cell lines also need to be fully validated.

In conclusion, we developed PSG-30, a prognostic gene signature for HCC LNM, to help identify HCC patients with LNM with poor prognosis. Through further regression model analysis of prognosis, *RAD54B* with the largest risk weight was selected as an independent prognostic risk factor. Through the systematic analysis of PSG-30-related subtypes and prognostic factor *RAD54B*, this work provides clues for the study of the pathogenesis of HCC LNM and the implementation of clinical individualized treatment.

## Data Availability

Data are available upon reasonable request. All data relevant to the study are included in the article or uploaded as supplementary information.

## Abbreviations

AUC: Area under curve
BP: Biological process
BRCA: Breast invasive carcinoma
CC: Cellular component
CDF: Cumulative distribution function
CHOL: Cholangiocarcinoma
CI: Confidence interval
CNV: Copy number variation
CRC: Colorectal carcinoma
DFS: Disease free survival
DSS: Disease-specific survival
EMT: Epithelial mesenchymal transition
GDSC: Drug sensitivity in cancer
GEO: Gene Expression Omnibus
HCC: Hepatocellular carcinoma
HR: Hazard ratio
ICI: Immune checkpoint inhibitor
LASSO: Least absolute shrinkage and selection operator
LIHC: Liver hepatocellular carcinoma
LNM: Lymph node metastasis
MAD: Median absolute deviation
MF: Molecular function
MSI: Microsatellite instability
OS: Overall survival
PFS: Progress free survival
PSG-30: Prognostic signature containing 30 genes
ROC: Receiver operating characteristic
TAMs: Tumor-associated macrophages
TCGA: The Cancer Genome Atlas
TMB: Tumor mutation burden
TME: Tumor microenvironment
TOM: Topological overlap matrix
TTP: Time to progression
WGCNA: Weighted gene coexpression network analysis.

## Supplementary Materials

Supplementary Materials Supplementary Figure 1: gene expression clustering analysis based on weighted gene coexpression network analysis (WGCNA) on paired samples from the GEO:GSE28248 dataset. (A and B) Selection of the soft-thresholding power (*β* = 6) with scale free topology model and mean connectivity. (C) Gene dendrogram and gene-module relationships obtained by hierarchical clustering of TOM-based dissimilarity. (D) The colored line below the gene dendrogram shows the three characteristic modules that are combined after being identified by the dynamic tree cutting algorithm (the grey module is defined as the gene set that cannot be assigned). Supplementary Figure 2: identification and phenotypic association analysis of LNM-related gene modules in HCC. (A) Clustering based on modular eigenvectors displayed significant discrimination between the identified modules. (B) A heatmap of the correlation between module and clinical traits of interest showed that the blue module had significant statistical significance in distinguishing HCC LNM (*P* = 7.4e − 3, *r* = 0.3). (C) A scatter plot of gene significance (GS) and module membership (MM) in blue module showed a significantly positive correlation (*P* = 2.9e − 6, *r* = 0.44); HCC LNM-related modules (blue) were further confirmed. (D) The coexpression network of 73 genes belonging to the blue module in the TCGA-LIHC data set. Supplementary Figure 3: differential gene analysis and gene function enrichment analysis of subtype 1 and subtype 4 separated by PSG-30. (A) Heatmap of differential gene expression among subtypes: the 50 upregulated genes (red) and 50 downregulated genes (blue) with the largest differential changes are shown here, and the green and red bands above the heatmap correspond to different subtypes. (B) A volcano plot of differentially expressed genes (fold change ≥ 1.5) between identified subtypes depicts the adjusted *P* value (−log10) vs. fold change (log2). (C) Functional enrichment: KEGG pathway and GO term enrichment results of differentially upregulated or downregulated genes among subtypes. Different colors represent the significance of differential enrichment terms, and the size of nodes reflects the number of enriched genes. Supplementary Figure 4: subtype verification based on independent HCC samples from GSE5975 microarray dataset. (A and B) Consistent clustering (*k* = 4) heatmap of 238 HCC samples and heatmap for PSG-30 related gene expression among the four subgroups. (C and D) Differential gene expression profile between subtype 2 and 4 presented with a volcano plot and a clustered heatmap (fold change ≥ 1.5). Different colors on the horizontal axis correspond to different subtype groups. (E) KEGG pathway and GO term enrichment results of differentially upregulated or downregulated genes among subtype 2 and 4. Different colors represent the significance of differential enrichment terms, and the size of nodes reflects the number of enriched genes. Supplementary Figure 5: verification of PSG-30's subgroup discrimination ability in CRC. (A) Heatmap of PSG-30 related gene expression in subtypes identified by consensus clustering (*k* = 4) in TCGA COADREAD samples (*N* = 620). Red represents high expression and blue represents low expression. (B–D) Enrichment analysis of DEGs between subtype 1 and 4. (E) Immune infiltration analysis of identified subgroups based on xCell algorithm. (F) The distribution of clinical characteristics in samples of different subtype groups. ^∗^*P* < 0.05, ^∗∗^*P* < 0.01, and ^∗∗∗^*P* < 0.001, Kruskal-Wallis test. Supplementary Figure 6: construction of LASSO regression model based on OS data and analysis of its correlation with immune cell infiltration. (A) Dynamic relationship between coefficients of selected features and regularization parameter *λ*. (B) The relationship between partial likelihood deviation and log (*λ*) is obtained by LASSO Cox regression model. (C) The composite diagram evaluates the relationship between risk score and survival time and survival status based on TCGA data set, in which the upper figure represents the scatter diagram of risk score from low to high, distinguishes high- and low-risk groups by color, and maps them to the middle and lower figures. The middle figure shows the scatter diagram distribution of survival time and survival state corresponding to high- and low-risk groups, and the states of “alive” and “dead” are distinguished by color. The lower figure represents the expression heatmap of the included prognostic factor genes in the sample. (D) The distribution of KM survival curve of the risk model in TCGA data set, in which the difference between groups is tested by log-rank test, and the mean time represents the median survival time in years. (E) The ROC curve and AUC value of the constructed risk model at the point of 1, 3, and 5 years. (F and G) Spearman correlation analysis between model risk score and immune score evaluated by TIMER (F) and quanTIseq (G). The horizontal axis represents the distribution of model risk score, the vertical axis represents the distribution of immune scores, and the right density curve represents the distribution trend of immune scores. The upper density curve is the distribution trend of model risk score. Supplementary Figure 7: construction of LASSO regression model based on DSS data and analysis of its correlation with TMB and MSI. (A) Dynamic relationship between coefficients of selected features and regularization parameter *λ*. (B) The relationship between partial likelihood deviation and log (*λ*) is obtained by LASSO Cox regression model. (C) The composite diagram evaluates the relationship between risk score and survival time and survival status based on TCGA dataset, in which the upper figure represents the scatter diagram of risk score from low to high, distinguishes high- and low-risk groups by color, and maps them to the middle and lower figures. The middle figure shows the scatter diagram distribution of survival time and survival state corresponding to high- and low-risk groups, and the states of “alive” and “dead” are distinguished by color. The lower figure represents the expression heatmap of the included prognostic factor genes in the sample. (D) The distribution of KM survival curve of the risk model in TCGA dataset, in which the difference between groups is tested by log-rank test, and the mean time represents the median survival time in years. (E) The ROC curve and AUC value of the constructed risk model at the point of 1, 3, and 5 years. (F and G) Correlation between independent prognostic molecule *RAD54B* and TMB (F)/MSI (G) in four subtypes. Supplementary Figure 8: prognostic analysis of *RAD54B* in samples with and without hepatitis virus. (A–D) KM plot of *RAD54B* expression in the hepatitis virus negative group with respect to OS, PFS, RFS, and DSS. (E–H) KM plot of *RAD54B* expression in the hepatitis virus-positive group with respect to OS, PFS, RFS, and DSS. Supplementary Figure 9-10: prognostic analysis of OS and RFS in HCC using the KM plot database for 70 genes selected. Genes with no prognostic significance in both OS and RFS have been deleted from display. And genes with one or both prognostic significance in OS and RFS were retained and displayed as KM plots. Supplementary Figure 11: the mutational landscape of PSG-30 and its clinical prognostic relevance. (A and B) The genomic changes of PSG-30 in samples from the 6 independent LIHC cohorts (*N* = 1026) of the cBioPortal platform and the distribution of mutation types in each cohort. (C and D) Comparison of the prognosis of PSG-30 mutant population and nonmutated population in OS and DFS. (E and F) Comparison of the incidence of LNM (Neoplasm Disease Lymph Node Stage American Joint Committee on Cancer Code) and vascular invasion between people with PSG-30 mutation and those without mutation. (G) The genome changes and mRNA expression of PSG-30 in the LIHC cohort samples (*N* = 360) derived from the TCGA dataset (Firehose Legacy). (H and I) Comparison of the prognosis of the PSG-30 genome-altered population and the unaltered population in DFS and OS. (J and K) The genomic changes of PSG-30 in 17 BRCA cohort samples (*N* = 7771) of the cBioPortal platform and the distribution of mutation types in each cohort. (L and M) The comparison of number of positive lymph nodes examined and the population distribution ratio between the BRCA samples with and without PSG-30 mutation. Supplementary Figure 12: coexpression analysis of biomarkers in TCGA LIHC dataset. (A) The expression correlation analysis of PSG-30 (horizontal coordinate) and HCC LNM-related free mRNAs (vertical coordinate). (B) The expression correlation analysis of PSG-30 (horizontal coordinate) and TAM/M2 macrophage markers (vertical coordinate). (C) The expression correlation analysis of TAM/M2 macrophage markers (horizontal coordinate) and HCC LNM-related free mRNAs (vertical coordinate).
