# Supplementary material for: Identification of a Prognostic Transcriptome Signature for Hepatocellular Carcinoma with Lymph Node Metastasis
Source: Oxid Med Cell Longev. 2022 Jul 6;2022:7291406. doi: 10.1155/2022/7291406 (PMC9279092; doi:10.1155/2022/7291406)
Supplement: Supplementary Materials — Supplementary Figure 1: gene expression clustering analysis based on weighted gene coexpression network analysis (WGCNA) on paired samples from the GEO:GSE28248 dataset. (A and B) Selection of the soft-thresholding power (β = 6) with scale free topology model and mean connectivity. (C) Gene dendrogram and gene-module relationships obtained by hierarchical clustering of TOM-based dissimilarity. (D) The colored line below the gene dendrogram shows the three characteristic modules that are combined after being identified by the dynamic tree cutting algorithm (the grey module is defined as the gene set that cannot be assigned). Supplementary Figure 2: identification and phenotypic association analysis of LNM-related gene modules in HCC. (A) Clustering based on modular eigenvectors displayed significant discrimination between the identified modules. (B) A heatmap of the correlation between module and clinical traits of interest showed that the blue module had significant statistical significance in distinguishing HCC LNM (P = 7.4e − 3, r = 0.3). (C) A scatter plot of gene significance (GS) and module membership (MM) in blue module showed a significantly positive correlation (P = 2.9e − 6, r = 0.44); HCC LNM-related modules (blue) were further confirmed. (D) The coexpression network of 73 genes belonging to the blue module in the TCGA-LIHC data set. Supplementary Figure 3: differential gene analysis and gene function enrichment analysis of subtype 1 and subtype 4 separated by PSG-30. (A) Heatmap of differential gene expression among subtypes: the 50 upregulated genes (red) and 50 downregulated genes (blue) with the largest differential changes are shown here, and the green and red bands above the heatmap correspond to different subtypes. (B) A volcano plot of differentially expressed genes (fold change ≥ 1.5) between identified subtypes depicts the adjusted P value (−log10) vs. fold change (log2). (C) Functional enrichment: KEGG pathway and GO term enrichment results of d [file S-figure-2.pdf › S-figure-2.pdf]

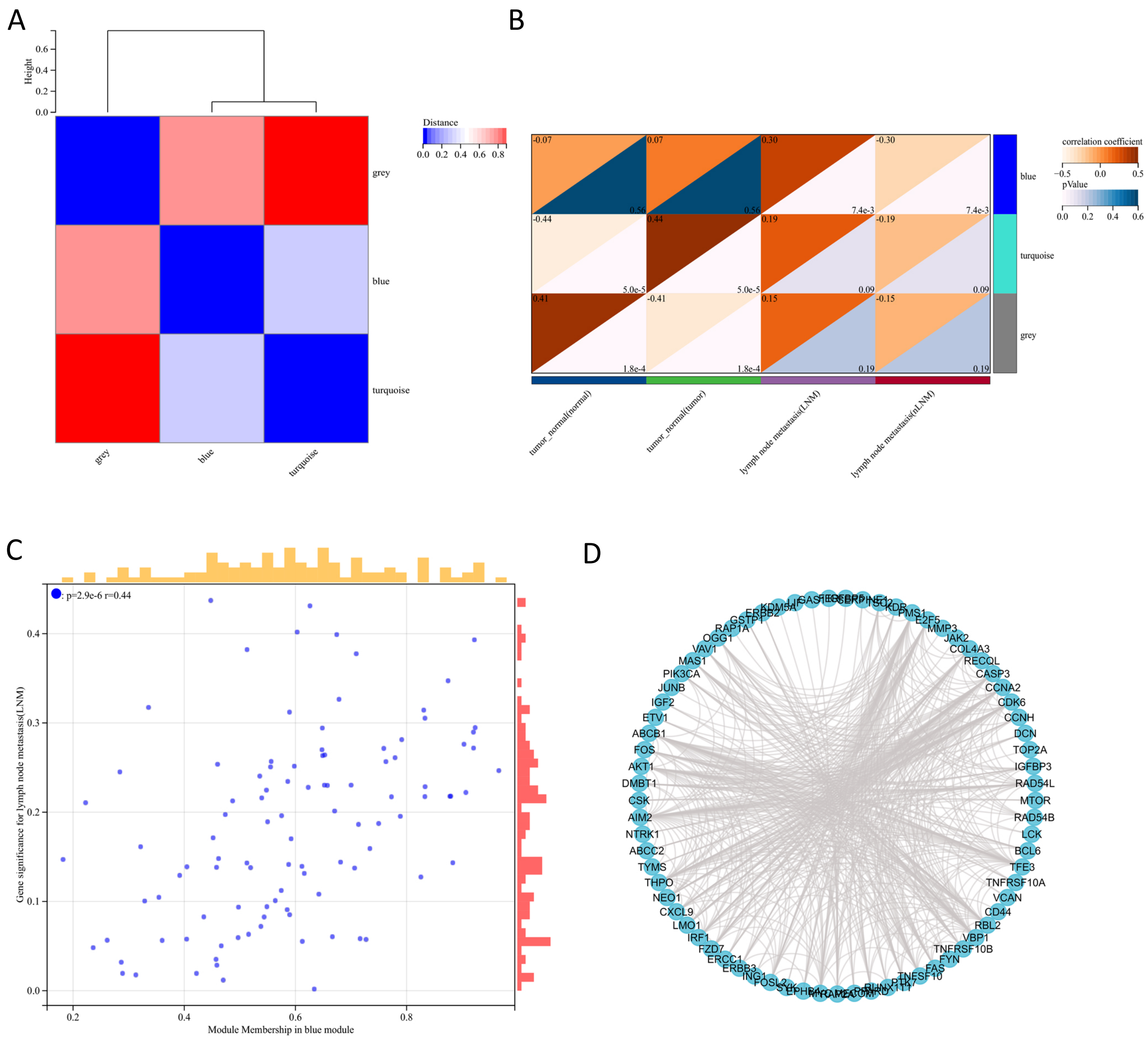

A
Height
0.0
0.2
0.4
0.6
Distance
0.0 0.2 0.4 0.6 0.8
grey
blue
turquoise
B
correlation coefficient
−0.5 0.0 0.5
pValue
0.0 0.2 0.4 0.6
-0.07
0.56
0.07
0.56
0.30
7.4e-3
-0.30
7.4e-3
-0.44
5.0e-5
0.44
5.0e-5
0.19
0.09
-0.19
0.09
0.41
1.8e-4
-0.41
1.8e-4
0.15
0.19
-0.15
0.19
blue
turquoise
grey
tumor_normal(normal)
tumor_normal(tumor)
lymph node metastasis(LNM)
lymph node metastasis(nLNM)
C
: p=2.9e-6 r=0.44
Gene significance for lymph node metastasis(LNM)
0.0
0.1
0.2
0.3
0.4
0.2
0.4
0.6
0.8
Module Membership in blue module
D
KDM5A
ERBB2
GSTP1
RAP1A
OGG1
VAV1
MAS1
PIK3CA
JUNB
IGF2
ETV1
ABCB1
FOS
AKT1
DMBT1
CSK
AIM2
NTRK1
ABCC2
TYMS
THPO
NEO1
CXCL9
LMO1
IRF1
FZD7
ERCC1
ERBB3
ING1
FOSL2
SYK
KDR
PMS1
E2F5
MMP3
JAK2
COL4A3
RECQL
CASP3
CCNA2
CDK6
CCNH
DCN
TOP2A
IGFBP3
RAD54L
MTOR
RAD54B
LCK
BCL6
TFE3
TNFRSF10A
VCAN
CD44
RBL2
VBP1
TNFRSF10B
FYN
FAS
TNFSF10
