# Supplementary material for: Identification of a Prognostic Transcriptome Signature for Hepatocellular Carcinoma with Lymph Node Metastasis
Source: Oxid Med Cell Longev. 2022 Jul 6;2022:7291406. doi: 10.1155/2022/7291406 (PMC9279092; doi:10.1155/2022/7291406)
Supplement: Supplementary Materials — Supplementary Figure 1: gene expression clustering analysis based on weighted gene coexpression network analysis (WGCNA) on paired samples from the GEO:GSE28248 dataset. (A and B) Selection of the soft-thresholding power (β = 6) with scale free topology model and mean connectivity. (C) Gene dendrogram and gene-module relationships obtained by hierarchical clustering of TOM-based dissimilarity. (D) The colored line below the gene dendrogram shows the three characteristic modules that are combined after being identified by the dynamic tree cutting algorithm (the grey module is defined as the gene set that cannot be assigned). Supplementary Figure 2: identification and phenotypic association analysis of LNM-related gene modules in HCC. (A) Clustering based on modular eigenvectors displayed significant discrimination between the identified modules. (B) A heatmap of the correlation between module and clinical traits of interest showed that the blue module had significant statistical significance in distinguishing HCC LNM (P = 7.4e − 3, r = 0.3). (C) A scatter plot of gene significance (GS) and module membership (MM) in blue module showed a significantly positive correlation (P = 2.9e − 6, r = 0.44); HCC LNM-related modules (blue) were further confirmed. (D) The coexpression network of 73 genes belonging to the blue module in the TCGA-LIHC data set. Supplementary Figure 3: differential gene analysis and gene function enrichment analysis of subtype 1 and subtype 4 separated by PSG-30. (A) Heatmap of differential gene expression among subtypes: the 50 upregulated genes (red) and 50 downregulated genes (blue) with the largest differential changes are shown here, and the green and red bands above the heatmap correspond to different subtypes. (B) A volcano plot of differentially expressed genes (fold change ≥ 1.5) between identified subtypes depicts the adjusted P value (−log10) vs. fold change (log2). (C) Functional enrichment: KEGG pathway and GO term enrichment results of d [file S-figure-5.pdf › S-figure-5.pdf]

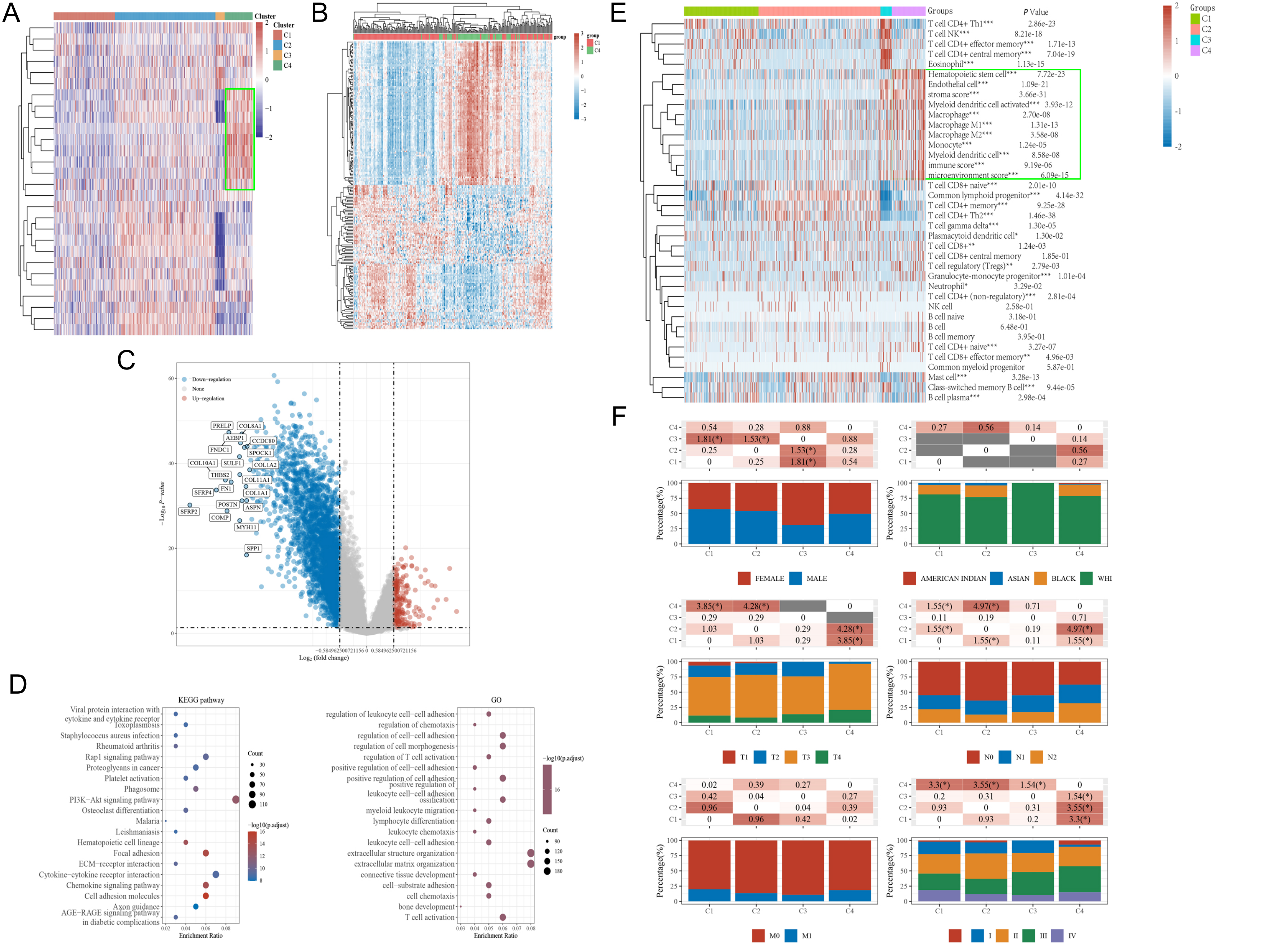
A
B
C
D
E
F
Cluster
C1
C2
C3
C4
group
Down-regulation
None
Up-regulation
Log2 (fold change)
−Log10 P-value
PRELP
COL8A1
AEBP1
CCDC80
FNDC1
SPOCK1
COL10A1
SULF1
COL1A2
THBS2
COL11A1
SFRP4
FN1
COL1A1
POSTN
ASPN
SFRP2
COMP
MYH11
SPP1
KEGG pathway
GO
Enrichment Ratio
Count
-log10(p.adjust)
Viral protein interaction with cytokine and cytokine receptor
Toxoplasmosis
Staphylococcus aureus infection
Rheumatoid arthritis
Rap1 signaling pathway
Proteoglycans in cancer
Platelet activation
Phagosome
PI3K−Akt signaling pathway
Osteoclast differentiation
Malaria
Leishmaniasis
Hematopoietic cell lineage
Focal adhesion
ECM−receptor interaction
Cytokine−cytokine receptor interaction
Chemokine signaling pathway
Cell adhesion molecules
Axon guidance
AGE−RAGE signaling pathway in diabetic complications
regulation of leukocyte cell−cell adhesion
regulation of chemotaxis
regulation of cell−cell adhesion
regulation of cell morphogenesis
regulation of T cell activation
positive regulation of cell−cell adhesion
positive regulation of cell adhesion
positive regulation of leukocyte cell−cell adhesion
ossification
myeloid leukocyte migration
lymphocyte differentiation
leukocyte chemotaxis
leukocyte cell−cell adhesion
extracellular structure organization
extracellular matrix organization
connective tissue development
cell−substrate adhesion
cell chemotaxis
bone development
T cell activation
Groups
P Value
T cell CD4+ Th1*** 2.86e-23
T cell NK*** 8.21e-18
T cell CD4+ effector memory*** 1.71e-13
T cell CD4+ central memory*** 7.04e-19
Eosinophil*** 1.13e-15
Hematopoietic stem cell*** 7.72e-23
Endothelial cell*** 1.09e-21
stroma score*** 3.66e-31
Myeloid dendritic cell activated*** 3.93e-12
Macrophage*** 2.70e-08
Macrophage M1*** 1.31e-13
Macrophage M2*** 3.58e-08
Monocyte*** 1.24e-05
Myeloid dendritic cell*** 8.58e-08
immune score*** 9.19e-06
microenvironment score*** 6.09e-15
T cell CD8+ naive*** 2.01e-10
Common lymphoid progenitor*** 4.14e-32
T cell CD4+ memory*** 9.25e-28
T cell CD4+ Th2*** 1.46e-38
T cell gamma delta*** 1.30e-05
Plasmacytoid dendritic cell* 1.30e-02
T cell CD8+** 1.24e-03
T cell CD8+ central memory 1.85e-01
T cell regulatory (Tregs)** 2.79e-03
Granulocyte-monocyte progenitor*** 1.01e-04
Neutrophil* 3.29e-02
T cell CD4+ (non-regulatory)*** 2.81e-04
NK cell 2.58e-01
B cell naive 3.18e-01
B cell 6.48e-01
B cell memory 3.95e-01
T cell CD4+ naive*** 3.27e-07
T cell CD8+ effector memory** 4.96e-03
Common myeloid progenitor 5.87e-01
Mast cell*** 3.28e-13
Class-switched memory B cell*** 9.44e-05
B cell plasma*** 2.98e-04
Percentage(%)
FEMALE
MALE
AMERICAN INDIAN
ASIAN
BLACK
WHI
T1
T2
T3
T4
N0
N1
N2
M0
M1
I
II
III
IV
